# Improved Catalytic Durability of Pt-Particle/ABS for H_2_O_2_ Decomposition in Contact Lens Cleaning

**DOI:** 10.3390/nano9030342

**Published:** 2019-03-03

**Authors:** Yuji Ohkubo, Tomonori Aoki, Satoshi Seino, Osamu Mori, Issaku Ito, Katsuyoshi Endo, Kazuya Yamamura

**Affiliations:** 1 Graduate School of Engineering, Osaka University, Suita, Osaka 565-0871, Japan; t-aoki@div1.upst.eng.osaka-u.ac.jp (T.A.); seino@mit.eng.osaka-u.ac.jp (S.S.); endo@upst.eng.osaka-u.ac.jp (K.E.); yamamura@prec.eng.osaka-u.ac.jp (K.Y.); 2 Menicon Co., Ltd., Kasugai, Aichi 487-0032, Japan; o-mori@menicon.co.jp (O.M.); issaku-ito@menicon.co.jp (I.I.)

**Keywords:** catalytic durability, nanoparticle, supported catalyst, radical reactions, platinum (Pt), H_2_O_2_ decomposition, contact lens cleaning

## Abstract

In a previous study, Pt nanoparticles were supported on a substrate of acrylonitrile–butadiene–styrene copolymer (ABS) to give the ABS surface catalytic activity for H_2_O_2_ decomposition during contact lens cleaning. Although the Pt-particle/ABS catalysts exhibited considerably high specific catalytic activity for H_2_O_2_ decomposition, the catalytic activity decreased with increasing numbers of repeated usage, which meant the durability of the catalytic activity was low. Therefore, to improve the catalytic durability in this study, we proposed two types of pretreatments, as well as a combination of these treatments before supporting Pt nanoparticles on the ABS substrate. In the first method, the ABS substrate was etched, and in the second method, the surface charge of the ABS substrate was controlled. A combination of etching and surface charge control was also applied as a third method. The effects of these pretreatments on the surface morphology, surface chemical composition, deposition behavior of Pt particles, and Pt loading weight were investigated by scanning electron microscopy (SEM), X-ray photoelectron spectroscopy (XPS), cross-sectional SEM, and inductively coupled plasma atomic emission spectroscopy (ICP-AES), respectively. Both etching and controlling the surface charge effectively improved the catalytic durability for H_2_O_2_ decomposition. In addition, the combination treatment was the most effective.

## 1. Introduction

The number of contact lens wearers is estimated to be approximately 140 million all over the world [1]. Contact lens materials have been improved based on demands from the wearers [2]. Contact lenses are divided into two types: disposable and extended wear. Extended-wear contact lenses can be used repeatedly, which offers long-term cost advantages. However, to prevent eye troubles, a repeatable-use-type contact lens requires daily cleaning and sterilization. Three methods are used to clean and sterilize contact lenses: boiling, and cleaning in either H_2_O_2_ or multipurpose solution (MPS). When cleaning with a MPS, a single solution plays the roles of cleaning, sterilizing, and preserving lenses. Thus, MPS cleaning is a simple method, and about 70% of contact lens wearers currently use an MPS to clean their lenses [3]. However, if contact lens wearers are not careful while using the MPS, eye troubles are likely to occur due to inadequate sterilization. Therefore, the number of wearers using H_2_O_2_ cleaning, which has higher sterilization performance, has gradually increased in recent years [3]. In H_2_O_2_ cleaning, a 35,000 ppm H_2_O_2_ solution is used to clean and sterilize contact lenses. Although the H_2_O_2_ solution exhibits high sterilizing performance, it involves the risk of eyes becoming bloodshot or painful, and can even lead to blindness if the H_2_O_2_ solution enters the eyes without decomposing. Thus, a Pt catalyst is used to promote H_2_O_2_ decomposition to lower the residual H_2_O_2_ concentration below 100 ppm [4]. A Pt film plated on an acrylonitrile–butadiene–styrene copolymer (ABS) container using electroless plating is usually used to catalyze H_2_O_2_ decomposition. Thus, each ABS container needs a Pt loading weight of 1.5 mg. However, Pt is an expensive material. In addition, the Pt-film/ABS container must be thrown away after repeated use for a month. Therefore, a technique that decreases the amount of Pt used to clean contact lenses is needed.

In a previous report [5], we proposed replacing the Pt film with Pt nanoparticles. Several methods can synthesize and immobilize metal nanoparticles, for example: impregnation [6,7,8], polyol [9,10,11], and sonolytic methods [12,13,14,15]. These methods have the disadvantages of high processing temperature, long processing time, nonuniform deposition of the metal ions, and low producibility. Therefore, we selected a radiolytic synthesis method that uses a high-energy electron beam (EB) to synthesize and immobilize Pt nanoparticles on the ABS container. This method is called the electron-beam irradiation reduction method (EBIRM), and it offers the advantages of a low processing temperature, short processing time, highly uniform deposition, and high producibility [16,17,18,19]. We successfully decreased the Pt loading weight from 1.5 mg/substrate for Pt-film/ABS to 5.9 μg/substrate for Pt-particle/ABS and synthesized a Pt-particle/ABS catalyst having considerably higher specific catalytic activity for H_2_O_2_ decomposition than the Pt-film/ABS catalyst. However, the catalytic activity of the Pt-particle/ABS catalyst decreased with increasing number of repeated uses, although the catalytic activity of the Pt-film/ABS catalyst did not change. The decrease in catalytic activity of the Pt-particle/ABS catalyst was caused by decreasing the Pt loading weight with increasing numbers of repeated uses, not by poisoning Pt particles [5]. This problem of catalytic durability for H_2_O_2_ decomposition remains unsolved. Thus, in this study, we attempted to improve the catalytic durability using two pretreatments before EB irradiation—etching and controlling the surface charge—and a combination of both. The effects of pretreatments on ABS surface, catalytic activity, and catalytic durability were investigated.

## 2. Results and Discussion

### 2.1. Effect of Pretreatment on ABS Substrate

**Figure 1:**
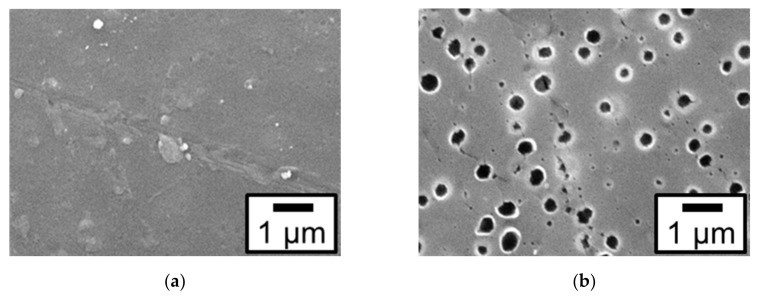
Scanning electron microscope (SEM) images of the surface of the acrylonitrile–butadiene–styrene copolymer (ABS) substrate not containing Pt particles before and after etching: (**a**) ABS-untreated and (**b**) ABS-Etch.

To examine the effect of etching on the surface morphology of the ABS substrate, etched ABS surfaces not containing Pt particles were observed using a scanning electron microscope (SEM). Figure 1 shows the SEM images of the surface of the ABS substrate before and after etching (the samples are hereafter labeled with their pretreatment and whether they contain Pt). Although no holes were present before etching, as shown in Figure 1a, many holes with a diameter of 100–500 nm appeared after etching, as shown in Figure 1b. The etching process was confirmed to dissolve butadiene rubber, thereby increasing the surface area of the ABS substrate.

**Figure 2:**
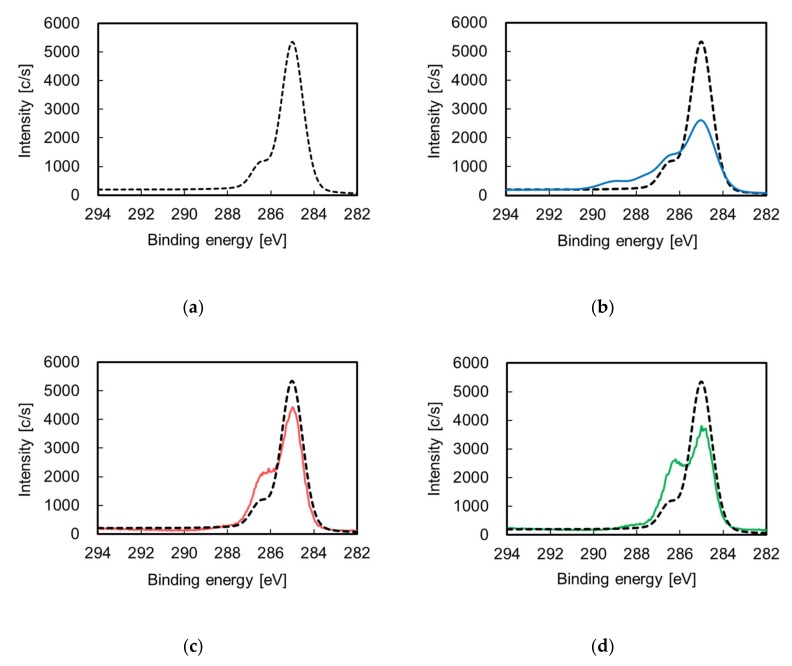
C1s-X-ray photoelectron spectroscopy (XPS) spectra of the ABS surface before (dotted lines) and after surface (solid colored lines): (**a**) ABS-untreated, (**b**) ABS-Etch, (**c**) ABS-Charge, and (**d**) ABS-Etch&Charge.

To examine the effects of etching and surface charge control on the chemical composition of an ABS substrate, the chemical compositions of the pretreated ABS surfaces not containing Pt particles were investigated using X-ray photoelectron spectroscopy (XPS). Figure 2a,b shows the C1s-XPS spectra of the surface of the ABS substrate before and after etching. When the ABS substrate was etched, the intensity of the peak indexed to C–H and C–C (285 eV) decreased whereas the intensity of the peaks indexed to C=O–O (289 eV), C=O (287.5 eV), C–N and C–O (286.5 eV) increased. These results indicate that etching not only dissolves butadiene rubber, but also introduces oxygen-containing functional groups. Figure 2a,c shows the C1s-XPS spectra of the surface of the ABS substrate before and after the surface charge control treatment. When the surface charge of the ABS substrate was controlled, the intensity of the peak indexed to C–H and C–C (285 eV) decreased, whereas that of the peaks indexed to C–N and C–O (286.5 eV) increased, indicating that the ABS surface was covered with surface charge controllers. When both etching and surface charge control were performed, the C1s-XPS spectrum for ABS-Etch&Charge was shaped similarly to that for ABS-Charge, as shown in Figure 2c,d, respectively. This similarity indicates that the ABS-Etch&Charge substrate was also covered with surface charge controllers.

**Figure 3:**
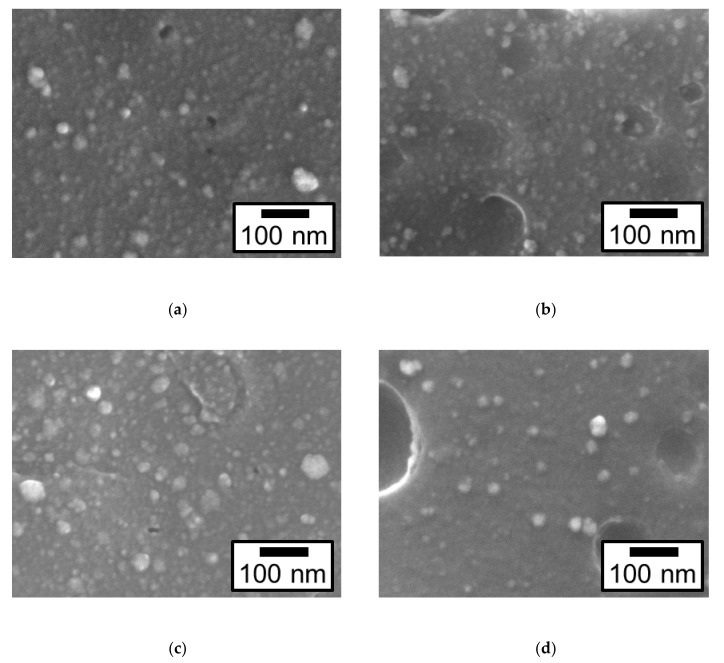
Field-emission (FE) SEM images of the surface morphology of four types of Pt/ABS samples: (**a**) Pt/ABS-untreated, (**b**) Pt/ABS-Etch, (**c**) Pt/ABS-Charge, and (**d**) Pt/ABS-Etch&Charge.

The effects of etching and surface charge control on the deposition behavior of Pt particles on four types of the ABS samples were examined. Figure 3 shows the field-emission (FE) SEM images of the surface morphology for the four types of Pt/ABS samples: Pt/ABS-untreated, Pt/ABS-Etch, Pt/ABS-Charge, and Pt/ABS-Etch&Charge. Main Pt particles with diameter of <20 nm and partial Pt particles with a diameter of 20–60 nm were observed on a surface of all four types of Pt/ABS samples. The size of the Pt particles was almost the same whether the surface was etched or its surface charge was controlled.

**Figure 4:**
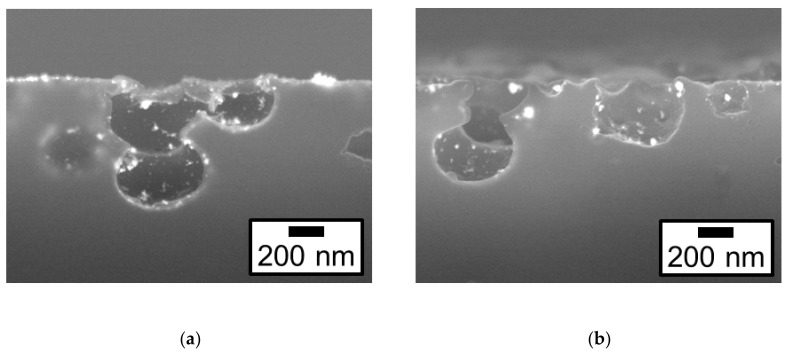
Cross-sectional backscattered electron images for etched samples: (**a**) Pt/ABS-Etch and (**b**) Pt/ABS-Etch&Charge.

Cross-sectional FE-SEM images confirmed where Pt particles were deposited on etched ABS samples. Figure 4 shows the cross-sectional backscattered electron images of the Pt/ABS-Etch and Pt/ABS-Etch&Charge samples. The Pt particles were observed both on the ABS surface and in the holes opened by etching. These results indicated that etching increased not only the specific surface area of ABS, but also the number of sites for Pt deposition. In addition, the deposition behavior of Pt particles in the holes was confirmed to be almost the same as that of Pt particles on the ABS surface.

**Figure 5:**
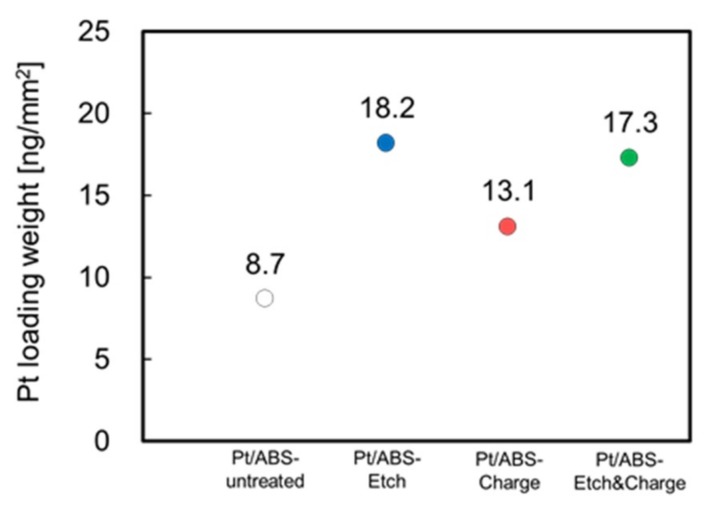
Pt loading weights of four types of Pt/ABS samples prepared using an electron-beam irradiation reduction method (EBIRM): Pt/ABS-untreated, Pt/ABS-Etch, Pt/ABS-Charge, and Pt/ABS-Etch&Charge.

The effects of etching and surface charge control on the Pt loading weight of Pt/ABS samples were also examined. Figure 5 shows the Pt loading weights of the four types of Pt/ABS samples. The Pt loading weights for the Pt/ABS-Etch, Pt/ABS-Charge, and Pt/ABS-Etch&Charge samples were higher than that for the Pt/ABS-untreated sample. This result indicated that both types of pretreatments and their combination increased the Pt loading weight. In addition, the Pt loading weights for the Pt/ABS-Etch and Pt/ABS-Etch&Charge samples were higher than that for the Pt/ABS-Charge sample. When an ABS substrate is etched, the butadiene rubber component dissolves, which results in a larger surface area. This larger surface area would increase the Pt loading weight because of the increase in sites for the immobilization of Pt nanoparticles. The Pt loading weight per unit area of the ABS substrate covered with an electroless-plated Pt film (Pt-film/ABS), which was calculated in the previous report [5], and the maximum Pt loading weight per unit area of Pt/ABS samples were 2240 and 18.2 ng/mm^2^, respectively. Thus, the amount of Pt consumed for the Pt/ABS samples prepared in this study was at least 120 times less than that for the Pt-film/ABS.

### 2.2. Catalytic Activity for H_2_O_2_ Decomposition

**Figure 6:**
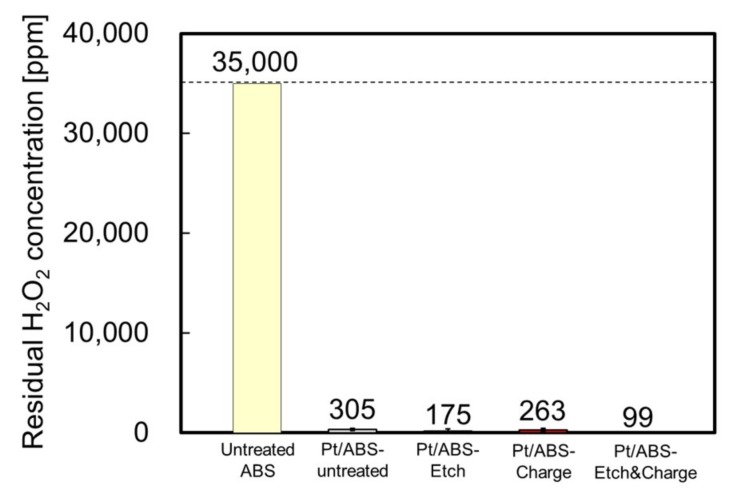
Catalytic activity of the untreated ABS and Pt/ABS samples with various pretreatment: residual H_2_O_2_ concentration after immersion for 360 min.

To evaluate the catalytic activity for H_2_O_2_ decomposition, the residual H_2_O_2_ concentration was measured after employing the four types of Pt/ABS samples. Thus, the untreated ABS, Pt/ABS-untreated, Pt/ABS-Etch, Pt/ABS-Charge, and Pt/ABS-Etch&Charge samples were immersed in a 35,000 ppm H_2_O_2_ solution for 360 min. The catalytic activities of the four types of Pt/ABS samples are compared in Figure 6. The untreated ABS sample did not decompose H_2_O_2_ at all within 360 min, whereas all the Pt-supported ABS samples significantly decreased the residual H_2_O_2_ concentration from 35,000 to less than 400 ppm. Moreover, the residual H_2_O_2_ concentrations for the Pt/ABS-Etch, Pt/ABS-Charge, and Pt/ABS-Etch&Charge samples became lower than that of the Pt/ABS-untreated sample. Therefore, the two types of pretreatments and their combination improved the catalytic activity for H_2_O_2_ decomposition. The difference in catalytic activity could be explained by the increase in Pt loading weight. The Pt/ABS-Etch&Charge sample exhibited the highest catalytic activity for H_2_O_2_ decomposition, successfully reaching the target value of 100 ppm.

### 2.3. Catalytic Durability in H_2_O_2_ Decomposition

**Figure 7:**
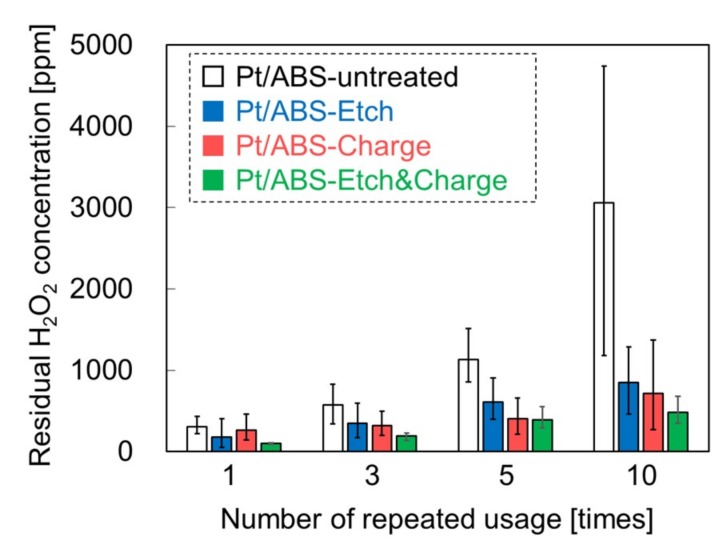
Catalytic durability of the Pt/ABS samples: relation between the number of repeated uses and the residual H_2_O_2_ concentration.

To examine the effects of etching and surface charge control on the catalytic durability, the relation between the number of repeated uses and residual H_2_O_2_ concentration was examined. Figure 7 shows the catalytic durability of the four types of Pt/ABS samples. After the Pt/ABS-untreated catalyst was used 10 times, the residual H_2_O_2_ concentration was 3056 ppm. This result suggests that much of the Pt remained on Pt/ABS-untreated, and more than 90% of H_2_O_2_ was decomposed after using it 10 times. However, this residual H_2_O_2_ concentration increased from 305 to 3056 ppm after repeated usage, thus demonstrating insufficient durability. For the pretreated Pt/ABS samples, the residual H_2_O_2_ concentrations after using Pt/ABS-Etch, Pt/ABS-Charge, and Pt/ABS-Etch&Charge samples 10 times were 851, 713, and 479 ppm, respectively. Thus, the residual H_2_O_2_ concentration decreased in the order: Pt/ABS-untreated > Pt/ABS-Etch > Pt/ABS-Charge > Pt/ABS-Etch&Charge. This result indicates that both etching and surface charge control effectively improved the catalytic activity for H_2_O_2_ decomposition. Moreover, the combination of etching and surface charge control was the most effective. However, the residual H_2_O_2_ concentration for Pt/ABS-Etch&Charge gradually increased with the number of repeated uses, suggesting that the catalytic durability was insufficient for use in practical applications, although the catalytic durability steadily improved upon etching and surface charge control. Therefore, the desorption of Pt nanoparticles must be further prevented.

## 3. Materials and Methods

### 3.1. Pretreatment and Synthesis of Pt-Particle/ABS

Pt nanoparticles were immobilized on an ABS substrate using EBIRM according to previous studies [16,20]. The methods for washing the ABS substrate and the radiolytic synthesis of Pt-particle/ABS samples were the same as those reported in the previous article [5]. The main difference between this and previous reports was the use of pretreatments to improve the catalytic durability for H_2_O_2_ decomposition.

A commercially available 1-mm-thick ABS sheet (2-9229-01, AS-ONE, Nishi-ku, Osaka, Japan) with dimensions of 20 mm × 15 mm × 1 mm was used as the ABS substrate. First, ABS substrates were sequentially washed with ethanol (99.5%, Kishida Chemical, Chuo-ku, Osaka, Japan) and pure water for 10 min each using an ultrasonic cleaner (USK-1R, AS-ONE). Then, they were dried using an N_2_ gun (99.99%, Iwatani Fine Gas, Amagasaki, Hyogo, Japan). Prior to immobilizing the Pt nanoparticles, the washed substrates were pretreated via either etching, surface charge control, or both. Table 1 shows the sample conditions and IDs.

A potassium permanganate solution (KMnO_4_, 0.2 M, Fujifilm Wako Pure Chemical, Chuo-ku, Osaka, Japan) and concentrated sulfuric acid (H_2_SO_4_; 97%, Kishida Chemical) were used for etching. An etching solution of molar ratio KMnO_4_/H_2_SO_4_ = 0.16/3.6 prepared according to patent [21] was used to dissolve butadiene rubber on the ABS surface. The ABS substrates were immersed in this etching solution for 20 min at room temperature. Then, the etched ABS substrates were washed with pure water for 10 min using an ultrasonic cleaner, followed by drying with an N_2_ gun.

**Figure 8:**
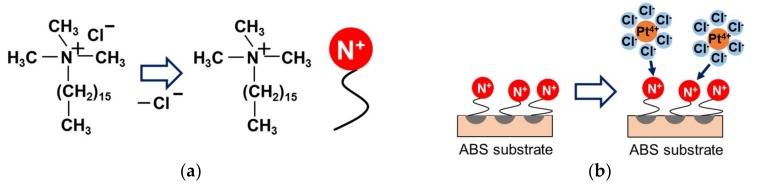
(**a**) Chemical formula of the surface charge controller (hexadecyltrimethylammonium chloride) and (**b**) schematic of electrostatic interactions between the surface charge controllers and Pt precursors (PtCl_6_^2−^).

Hexadecyltrimethylammonium chloride (Condiriser FR Conc, Okuno Chemical Industries, Chuo-ku, Osaka, Japan) was used to control surface charge, as shown in Figure 8. The hydrocarbon part adsorbs onto the ABS surface, whereas the ammonium end group modifies the surface charge of the ABS surface to be positive. Hexachloroplatinic acid hexahydrate (H_2_PtCl_6_·6H_2_O; 98.5%, Wako Pure Chemical Industries) was used as the Pt precursor. H_2_PtCl_6_·6H_2_O becomes PtCl_6_^2−^ in aqueous solution—that is, it becomes negatively charged—which is why Condiriser FR was selected. A 5% *v*/*v* solution of Condiriser FR was prepared, and ABS substrates were immersed in this surface charge controller solution for 5 min at 40 °C while stirring at 300 rpm using a magnetic hot stirrer (RCT Basic, IKA, Staufen, Baden-Württemberg, Germany) and a PTFE stirring bar. Excess surface charge controllers were washed away from the ABS substrates with pure water for 20 s. Then, the substrates were dried naturally at room temperature.

**Figure 9:**
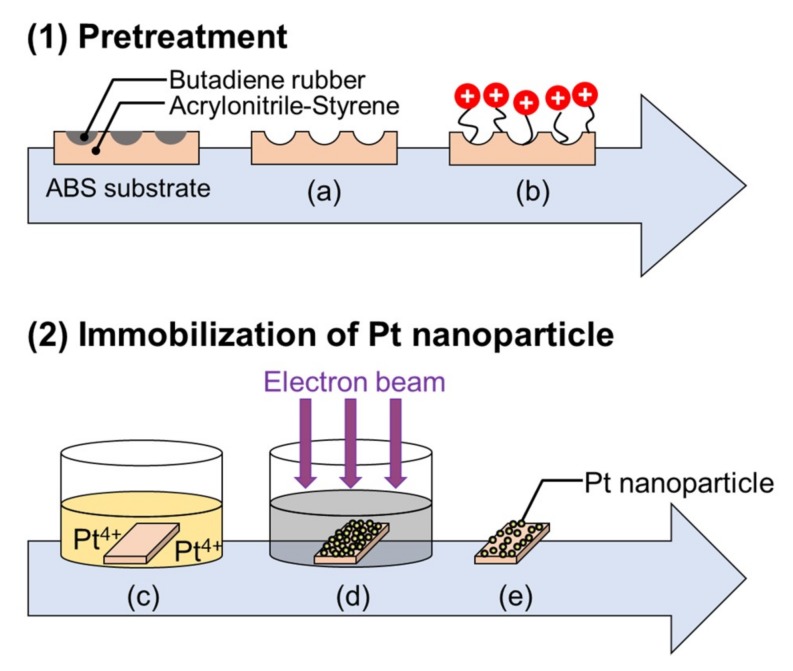
Schematic of the processes for pretreating and preparing a Pt-particle/ABS-Etch&Charge sample using an EBIRM through pretreatment: (**a**) KMnO_4_/H_2_SO_4_ etching to dissolve butadiene rubber on the ABS surface; (**b**) surface charge control; (**c**) immersion of the pretreated ABS substrate in the Pt precursor solution; (**d**) irradiation with an electron beam; and (**e**) removing it from the solution, washing using an ultrasonic cleaner, and drying by blowing with N_2_ gas.

To immobilize the Pt nanoparticles, 4 mM solutions of H_2_PtCl_6_ were separately prepared in cylindrical polystyrene (PS) containers (diameter = 33 mm and height = 16 mm), and 2-propanol (IPA; 99.7%, Kishida Chemical) was added to the solutions to be controlled at 1% *v*/*v*. Then, the pretreated ABS substrates were immersed in the Pt precursor solutions. These PS containers with the Pt precursor solutions and the pretreated ABS substrates were then irradiated for 7 s with a high-energy EB of 4.8 MeV using the Dynamitron^®^ accelerator at SHI-ATEX Co. Ltd., in Osaka, Japan. After EB irradiation, the substrates were removed from the solution and were washed with pure water using an ultrasonic cleaner for 10 min to remove the unsupported Pt nanoparticles. Finally, they were dried using the N_2_ gun. Figure 9 schematically shows the entire process. 

### 3.2. Characterization

To confirm that butadiene rubber dissolved from the ABS surface, the ABS surface was observed before and after etching using a SEM (JCM-6000, JEOL, Akishima, Tokyo, Japan) at an accelerating voltage of 10 kV. Prior to observation, a thin layer of Au was sputtered on the ABS surfaces using a Smart Coat DII-29010SCTR (JEOL) to prevent from electrostatic charge buildup during SEM observation.

To investigate the effects of etching and surface charge control on the chemical composition of the ABS surface, XPS measurement was performed using a Quantum 2000 (Ulvac-Phi, Chigasaki, Kanagawa, Japan) attached to an Al-*K*α source at 15 kV. The area of X-ray irradiation was ⌀100 μm, the pass energy was 23.50 eV, and the step size was 0.05 eV. The XPS spectra were recorded at take-off angles of 45°. A low-speed EB and an Ar ion beam were irradiated on the measured samples during the XPS measurement to neutralize their charges.

To investigate the deposition behavior of the Pt particles on the ABS surface, the ABS surfaces of the four types of Pt/ABS samples were observed using a FE-SEM (JSM-7800F, JEOL) at an accelerating voltage of 8 kV. Prior to observation, Os was coated on the Pt/ABS surfaces via plasma chemical vapor deposition using an Osmium Coater HPC-20 (VACUUM DEVICE, Mito, Ibaragi, Japan) to prevent electrostatic charge buildup during FE-SEM observation. Cross-sectional samples of Pt/ABS-Etch and Pt/ABS-Etch&Charge were prepared using a cross section polisher (IB-09020CP, JEOL) with a broad Ar ion beam source. Cross-sectional backscattered electron images were also obtained using the same FE-SEM (JSM-7800F, JEOL).

To measure the Pt loading weight on the Pt/ABS samples, inductively coupled plasma atomic emission spectrometry (ICP-AES; ICPE-9000, Shimadzu, Kyoto, Kyoto, Japan) was utilized. Pt nanoparticles on the Pt/ABS substrates were dissolved using aqua regia (volume ratio of HCl/HNO_3_ = 3/1). Then, the diluted aqua regia solutions were sprayed into a plasma torch in the ICPE-9000. The amount of Pt in the Pt/ABS samples was calculated from a calibration curve of a Pt standard solution (1000 ppm, Wako Pure Chemical Industries), as shown in the Supporting Information of the previous report [5].

H_2_O_2_ decomposition is accelerated by the platinum catalysts, then water and oxygen gas are generated, as shown in Equation (1):

2H_2_O_2_ → 2H_2_O + O_2_ (1)

The generation of O_2_ bubbles were observed during the H_2_O_2_ decomposition test in the present study as well as the previous study [5]. It was clear that H_2_O_2_ decomposition was accelerated by the Pt/ABS samples. To evaluate the catalytic activity for H_2_O_2_ decomposition, the residual H_2_O_2_ concentration was measured after immersing the Pt/ABS samples in a 35,000 ppm diluted solution of H_2_O_2_ (30% *w*/*w*, Kishida Chemical) at 25 °C for 360 min in an incubator (i-CUBE FCI-280, AS-ONE). In the previous study, a H_2_O_2_ decomposition curve was obtained by collecting the data of the residual H_2_O_2_ concentration with different H_2_O_2_ decomposition times of 2, 5, 10, 20, 30, 60, 120, 240, and 360 min, and it was confirmed that the residual H_2_O_2_ concentration steadily decreased with increasing H_2_O_2_ decomposition times [5]. Therefore, in the present study, the residual H_2_O_2_ concentration was measured after immersion of the Pt/ABS samples for only 360 min. The method for measuring the H_2_O_2_ concentration was the same that reported in the previous article [5]. A 5% *w*/*w* diluted solution of titanium sulfate (Ti(SO_4_)_2_; 30% *w*/*w*, Wako Pure Chemical Industries) was added to the H_2_O_2_ solution to color the H_2_O_2_ solution. Then, the optical absorbance of the colored H_2_O_2_ solution was measured using a deuterium-halogen and tungsten lamp (DH-2000, Ocean Optics, Largo, FL, USA), fiber multichannel spectrometer (HR-4000, Ocean Optics), and optical fiber (P600-1-UV/VIS, Ocean Optics). The absorbance at 407 nm was used to calculate the residual H_2_O_2_ concentration from the calibration curve, as shown in the Supporting Information of the previous report [5].

To evaluate the catalytic durability for H_2_O_2_ decomposition, the residual H_2_O_2_ concentration was measured after Pt/ABS samples were repetitively used 1, 3, 5, and 10 times.

## 4. Conclusions

We prepared four types of Pt/ABS catalysts with EBIRM and investigated the effects of two types of pretreatments—etching, surface charge control, and the combination of both—on the ABS surface, catalytic activity, Pt loading weight, and durability of these catalysts. Etching increased the Pt loading weight because of the increase in surface area of the ABS substrate, which in turn increased the catalytic activity. Etching also increased the catalytic durability, which could be attributed to the holes created by etching, which partially prevented Pt particles from detaching from the ABS surface. Surface charge control increased the Pt loading weight, which increased both the catalytic activity and durability. These improvements could be explained by electrostatic interactions between the Pt nanoparticles and surface charge controllers on the ABS substrate. The effects of etching on the Pt loading weight and catalytic activity were larger than those of the surface charge control. In contrast, the effect of surface charge control on the catalytic durability was higher than that of etching. Finally, the combination of etching and surface charge control most effectively improved both the catalytic activity and durability. Thus, we successfully improved the catalytic durability through either etching, surface charge control, or both before EB irradiation. Although the catalytic durability was insufficient for cleaning contact lenses in practical applications, these pretreatments would be useful for improving the adhesion between metal nanoparticles and resin substrates or microparticles except in severe conditions such as in H_2_O_2_ solution.

## Figures and Tables

**Table 1:** Sample condition and IDs.

Sample ID	Etching	Surface Charge Modification	EBIRM (EB Irradiation)
ABS-untreated	―	―	―
ABS-Etch	〇	―	―
ABS-Charge	―	〇	―
ABS-Etch&Charge	〇	〇	―
Pt/ABS-untreated	―	―	〇
Pt/ABS-Etch	〇	―	〇
Pt/ABS-Charge	―	〇	〇
Pt/ABS-Etch&Charge	〇	〇	〇
